# Preoperative alkaline phosphatase‐to‐platelet count ratio as a prognostic factor for hepatocellular carcinoma with microvascular invasion

**DOI:** 10.1002/cam4.6368

**Published:** 2023-07-26

**Authors:** Yongxin Zhang, Bin Zhang, Lianggeng Gong, Liangxia Xiong, Xuehong Xiao, Chao Bu, Zhiying Liang, Liangcai Li, Binghang Tang, Yangbai Lu

**Affiliations:** ^1^ Department of MR Zhongshan City People's Hospital Zhongshan China; ^2^ Department of Radiology The First Affiliated Hospital of Jinan University Guangzhou China; ^3^ Department of Medical Imaging Center The second affiliated Hospital of Nanchang University Nanchang China; ^4^ Department of Radiology The Seventh Affiliated Hospital Sun Yat‐Sen University Shenzhen China; ^5^ Department of Radiology, State Key Laboratory of Oncology in South China, Collaborative Innovation Center for Cancer Medicine, Guangdong Key Laboratory of Nasopharyngeal Carcinoma Diagnosis and Therapy Sun Yat‐sen University Cancer Center Guangzhou China; ^6^ Department of CT Zhongshan City People's Hospital Zhongshan China; ^7^ Department of Urology Zhongshan City People's Hospital Zhongshan China

**Keywords:** aspartate aminotransferase to platelet ratio, disease‐free survival, hepatocellular carcinoma, overall survival

## Abstract

**Objectives:**

The association between platelet status and hepatocellular carcinoma (HCC) prognoses remains controversial. Herein, we aimed to clarify the prognostic value of multiple platelet‐related biomarkers, including platelet count, platelet/lymphocyte ratio (PLR), aspartate aminotransferase to platelet ratio index (APRI), and alkaline phosphatase‐to‐platelet count ratio index (APPRI) in HCC with microvascular invasion (MVI) after curative resection or liver transplantation.

**Materials and Methods:**

A retrospective review of 169 patients with solitary HCC and MVI who underwent resection or liver transplantation between January 2015 and December 2018 was conducted. Preoperative clinical, laboratory, pathologic, and imaging data were collected and analyzed. Overall survival (OS) and disease‐free survival (DFS) were defined as the clinical endpoints. Univariate and multivariate Cox proportional hazards regression analyses were conducted to investigate potential predictors of DFS and OS.

**Results:**

Multivariate Cox regression analyses revealed that maximum tumor diameter, poor cell differentiation, and APPRI were independent predictors of DFS; while poor cell differentiation, APRI, APPRI, prothrombin time, and alpha‐fetoprotein were independent prognostic factors for OS. The 1‐, 3‐, and 5‐year DFS rates were 66.90%, 48.40%, and 37.40% for patients with APPRI ≤0.74 and 40.40%, 24.20%,and 24.20% for patients with APPRI>0.74. The corresponding rates of OS over 1, 3, and 5 years were 92.40%, 88.10% and 77.70%, and 72.30%, 38.20%, and 19.10%, respectively. The DFS and OS rates of patients whose APPRI was more than 0.74 were substantially lower than those of patients whose APPRI was less than or equal to 0.74 (*p* = 0.002 and *p* < 0.001, respectively).

**Conclusion:**

Elevated preoperative APPRI is a noninvasive, simple, and easily assessable parameter linked to poor prognosis in individuals with single HCC and MVI after resection or liver transplantation.

## 1 INTRODUCTION

Liver cancer is one of the leading causes of cancer‐related mortality around the world. Hepatocellular carcinoma (HCC) is the most common primary cancer of the liver.^1^ Liver transplantation and hepatectomy are both viable treatment strategies for treating HCC.^2^ Nevertheless, owing to the high rate of tumor recurrence, the long‐term prognosis is unsatisfactory.^3^, ^4^

The risk factors of HCC's outcomes have been identified, and include liver cirrhosis, tumor stage, tumor differentiation, tumor size, alpha‐fetoprotein (AFP), resection margin status as well as a microvascular invasion (MVI).^5^, ^6^, ^7^ Among these prognostic indicators, the MVI status is a well‐known major prognostic factor of HCC after surgery^8^; however, the survival of patients with MVI in the long term varies significantly. To date, studies have seldom assessed the prognosis of HCC patients with MVI specifically after surgery, and thus post‐surgical long‐term prognostic factors of these patients remain to be elucidated. Considering that MVI itself is a well‐known risk factor and is associated with multiple clinical variables, it is unclear whether the already identified risk factors for HCC remain suitable for this subgroup of patients or not. Zhou et al. showed that Edmondson‐Steiner grade and hepatitis B surface antigen positivity as potential indicators of poor prognosis and recurrence by examining 62 patients with HCC and MVI after curative hepatic resection.^9^ Xu et al. identified age, gross vascular invasion, tumor size, Glisson's capsule invasion, and nodule number as independent risk factors for the survival of HCC patients with MVI.^10^ These findings are meaningful but far from enough. In a clinical setting, the identification of prognostic biomarkers in subgroups of patients with HCC and MVI may be beneficial for precision management for closer monitoring and aggressive treatment strategy.

Previous studies have proven that platelets has a vital role in the growth, invasion, and angiogenesis of HCC.^11^, ^12^, ^13^ The association between platelet status and survival in HCC patients has been extensively explored. Nevertheless, previous results have no consensus and it seems that low preoperative platelet count has adverse effects on the survival of HCC patients after hepatic resection^14^, ^15^ but HCC individuals with thrombocytosis demonstrate a remarkably shorter survival duration.^16^, ^17^, ^18^ Several other platelet‐derived indices, including the aspartate aminotransferase to platelet ratio index (APRI) and platelet/lymphocyte ratio (PLR), are also related to early recurrence or poor survival in HCC patients.^19^, ^20^, ^21^, ^22^ However, scarce data have shown the role of alkaline phosphatase/platelet count ratio index (APPRI) in the evaluation of the prognosis of HCC patients. Moreover, very few studies have evaluatedthe above‐mentioned platelet‐based parameters together. The prognostic significance of platelet‐based inflammatory parameters in HCC patients has gained increasing interest. In this present study, we developed a risk classification system for patients with HCC with MVI after undergoing liver transplantation or resection by assessing the prognostic significance of platelet‐based biomarkers in this population.

## 2 MATERIALS AND METHODS

### 2.1 Patient cohort

**FIGURE 1:**
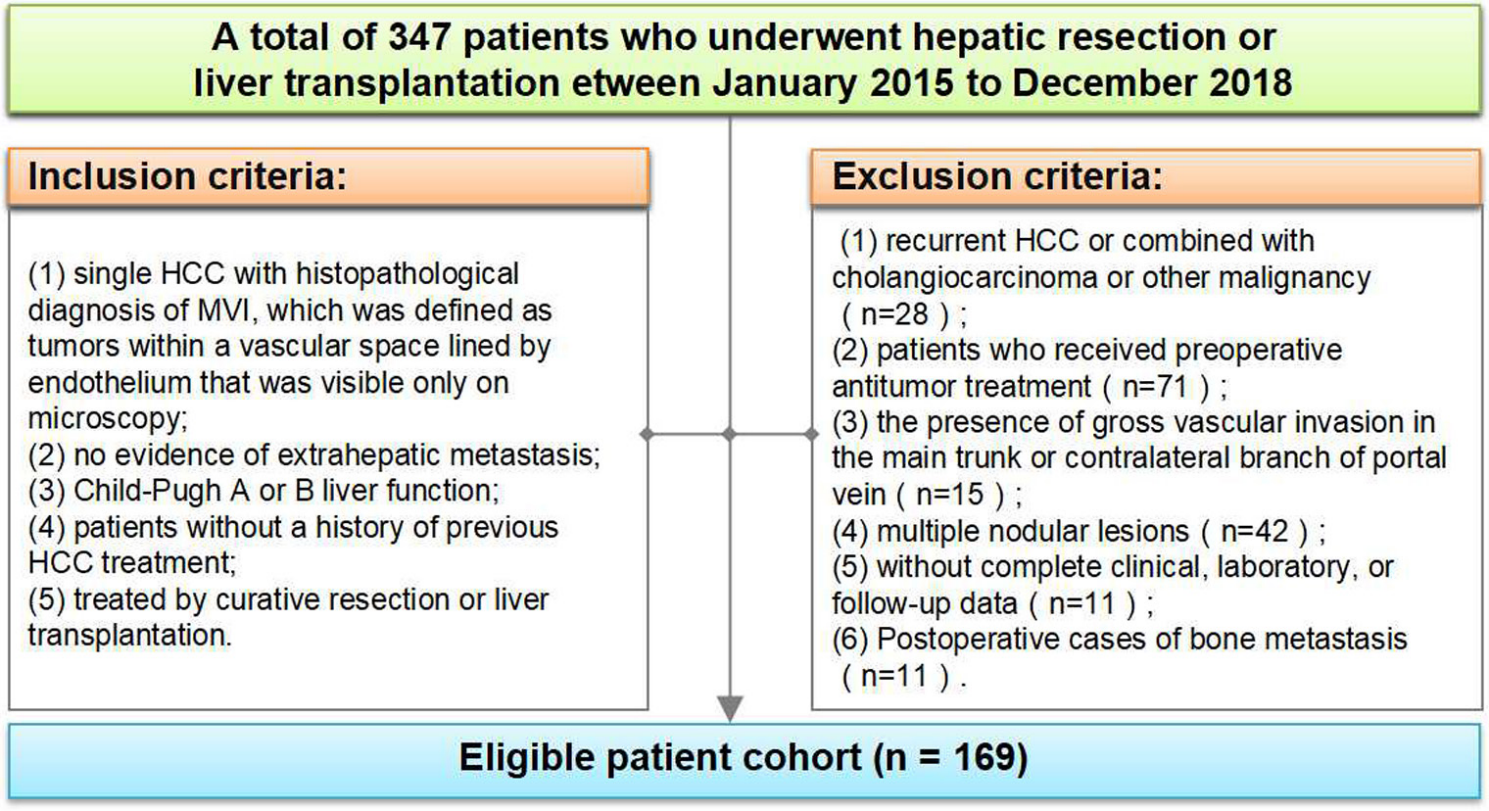
Flowchart of patient selection.

The institutional review board granted its approval for this retrospective study and waived the requirement for informed consent given the design. From January 2015 to December 2018, 347 patients with HCC and MVI underwent liver transplantation or curative resections in our hospital. Inclusion criteria were as follows: (1) solitary HCC with histopathological diagnosis of MVI defined as only microscopically detectable malignancies inside a vascular space lined by endothelium; (2) no evidence of extrahepatic metastasis; (3) liver function was assessed as Child‐Pugh A or B; (4) patients without a history of previous HCC treatment, and (5) those treated by curative resection or liver transplantation. When the histological investigation revealed no sign of a tumor and a negative resection (R0) margin, it was considered a curative resection. The exclusion criteria were as follows: (1) recurrent HCC or concurrent cholangiocarcinoma or other tumors; (2) patients who received preoperative antitumor treatment; (3) the presence of gross vascular invasion in the main trunk or contralateral branch of the portal vein; (4) multiple nodular lesions; (5) absence of complete laboratory, clinical, or follow‐up information, and (6) postoperative cases of bone metastasis. Finally, 169 HCC patients with MVI were included, of which, 159 (94.1%) underwent curative resection and 10 (5.9%) underwent liver transplantation. Figure 1 illustrates the schematic of patient inclusion in this study.

### 2.2 Clinicopathological and radiological variables

The clinicopathologic and radiologic variables were collected from the electronic medical records and preoperative MRI/CT scans, including sex, age, tumor stage, tumor differentiation, the presence of cirrhosis, Child‐Pugh grade, maximum tumor diameter (cm), number of segments involved, hepatitis B surface antigen/hepatitis be antigen (HbsAg/HbeAg), glutamic‐pyruvic transaminase (ALT), glutamic oxalacetic transaminase (AST), albumin, globulin, total bilirubin (TBIL), platelet count, platelet to lymphocyte ratio (PLR), lymphocyte to monocyte ratio (LMR), neutrophil to lymphocyte ratio (NLR), alkaline phosphatase‐to‐platelet count ratio index (APPRI), aspartic acid aminotransferase/platelet ratio index (APRI), prothrombin time (PT), serum alpha‐fetoprotein (AFP), and Albumin‐Bilirubin (ALBI).

Note that tumor differentiation was identified by the Edmondson‐Steiner system. Tumors were staged according to the American Joint Committee on Cancer/tumor‐node‐metastasis system (version 8).^23^ We calculated the preoperative Child score for each patient based on preoperative data, which involved hepatic encephalopathy, ascites, TBIL, PT, and albumin. Each item was scored 1–3 according to severity, and the grading criteria are as follows: Child‐Pugh class A, 5–6 points; Child‐Pugh class B, 7–9 points; Child‐Pugh class C, 10–15 points. ALBI = 0.66 x log10 TBIL−0.085 x ALB. The calculation formulas of these serum inflammatory markers were as follows:

(1) $$\begin{array}{c} \mathrm{PLR}=\text{Platelet Count}\;\left(\times 10^{9}/\text{L}\right)/\\ \text{Lymphocyte Count}\;\left(\times 10^{9}/\text{L}\right) \end{array}$$

(2) $$\begin{array}{c} \mathrm{LMR}=\text{Lymphocyte Count}\;\left(\times 10^{9}/\text{L}\right)/\\ \text{Monocyte Count}\;\left(\times 10^{9}/\text{L}\right) \end{array}$$

(3) $$\begin{array}{c} \mathrm{NLR}=\text{Neutrophil Count}\;\left(\times 10^{9}/\text{L}\right)/\\ \text{Lymphocyte Count}\;\left(\times 10^{9}/\text{L}\right) \end{array}$$

(4) $$\begin{array}{c} \text{APRI}=[(\mathrm{AST}\;\text{value}\left(\text{U}/\text{L}\right)/\\ \text{upper limit of normal value}\;\left(\text{U}/\text{L}\right)/\\ \mathrm{PLT}\;\left(\times 10^{9}/\text{L}\right)]\times 100 \end{array}$$

(5) $$\text{APPRI}=\left(\mathrm{ALP}\;\text{value}/\text{platelets count}\right)\times 10^{10}/\text{U}$$

### 2.3 Follow‐up surveillance after surgery

Clinical examinations and serum AFP level, along with chest and abdominal ultrasonography, CT or MRI scan of the chest and abdomen at 1 month postoperatively and 3 months postoperatively for the first 3 years, and every 6 months after that were all included in the postoperative follow‐up after patients were discharged from the hospital. Disease‐free survival (DFS) was the primary endpoint and was calculated from the time of surgery to the first relapse of malignancy. The identification of tumor recurrence was validated by further imaging and/or biopsy. Patient records with no evidence of tumor recurrence at the time of death or the end of follow‐up were censored. Overall survival (OS) was the secondary endpoint and was calculated as the time between the date of surgery and the date of death from any cause, or the date of the last contact with the patient if alive.

### 2.4 Statistical analysis

Continuous variables are expressed as means and standard deviations or medians and interquartile ranges. We used the *t*‐test or the Mann–Whitney *U* test for comparative analyses, as appropriate. Chi‐square and Fisher's exact tests were used to compare the categorical variables presented as percentages and numbers. The optimal cutoff values for the variables were determined using the receiver operating characteristic (ROC) curve analysis with the maximum Youden index method. By conducting Cox proportional hazards regression analysis, we identified the independent risk factors for DFS and OS. Univariate Cox model variables with *p* < 0.10 were subjected to the multivariate Cox analysis. A backward selection strategy was employed to choose the variables in the final model. In the multivariate Cox model, the independent risk factors were those with *p* < 0.05. Hazard ratios (HRs) were reported for the corresponding 95% confidence intervals (CIs). Survival differences between groups were compared using Kaplan–Meier curves and the log‐rank test. The statistical significance was set at *p* < 0.05. All analyses were done using SPSS 22.0 software (IBM Corp.).

## 3 RESULTS

### 3.1 Patient characteristics

The mean age of the 169 patients was 52.4 years ± 12.2. A total of 10 (5.9%) had stage IA disease while 159 (94.1%) had stage IB disease. Of the 169 patients, 22 (13%) were females and 147 (87%) were males. The mean and median tumor sizes were 7.0 and 6.7 cm, respectively. The median follow‐up time was 25 months (range 1–59 months). The median DFS was 17 months (range 1–58 months). Tumor recurrence occurred in 72 out of 169 (43%) patients, including cases of intrahepatic recurrence (*n* = 60), extrahepatic recurrence (*n* = 40), and both (*n* = 28).

### 3.2 Univariate and multivariate analyses for DFS and OS

On univariate analysis of DFS, maximum tumor diameter (*p* = 0.054), number of segments involved (*p* = 0.087), poor cell differentiation (*p* = 0.054), albumin (40‐50 g/L) (*p* = 0.020), NLR (*p* = 0.017), APRI (*p* = 0.005), APPRI (*p* = 0.003), Child‐Pugh grade (*p* = 0.074), and ALBI (*p* = 0.080) with *p* values less than 0.10 were enlisted in a multivariate Cox regression model (Table 1). All other variables were not independently associated with DFS in this model except for maximum tumor diameter (HR = 1.637; 95% CI: 1.075–2.493; *p* = 0.022), poor cell differentiation (HR = 1.678; 95% CI: 1.113–2.531; *p* = 0.014) and APPRI >0.74 (HR = 2.093; 95% CI:1.359–3.223; *p* = 0.001) (Table 1). Results of the univariate analysis for OS showed that poor cell differentiation (*p* = 0.075), albumin (*p* = 0.002 and *p* = 0.008), platelet count>320 × 10^9^/L (*p* = 0.095), LMR (*p* = 0.087), APPRI (*p* < 0.001), APRI (*p* < 0.001), PT (*p* = 0.001), AFP > 200 ng/m(*p* = 0.007), Child‐Pugh grade (*p* < 0.001) and ALBI (*p* = 0.001) (all *p* < 0.10) (Table 2). Prognostic factors for OS in this model included poor cell differentiation (HR = 1.945; 95% CI:1.012–3.738; *p* = 0.046), preoperative APPRI>0.74 (HR = 3.744; 95% CI:1.669–8.400; *p* = 0.001), preoperative APRI >0.79 (HR = 2.824; 95% CI: 1.275–6.255; *p* = 0.011), preoperative PT > 13.5 s (HR = 3.858; 95% CI:1.782–8.354; *p* = 0.001), and preoperative AFP > 200 ng/mL (HR = 3.763; 95% CI:1.565–9.052; *p* = 0.003) (Table 2). Therefore, in patients with HCC and MVI, only preoperative APPRI and poor cell differentiationwere were independent risk factors for both OS and DFS.

**TABLE 1:** Multivariate and univariate Cox analyses of risk factors for disease‐free survival (DFS) in patients with hepatocellular carcinoma with microvascular invasion.

	Univariate		Multivariate	
	HR (95% CI)	*p*	HR (95% CI)	*p*
Sex				
Female	Reference		NA	NA
Male	1.287 (0.668–2.478)	0.451	NA	NA
Age, years				
≤50	Reference			
>50	0.893 (0.596–1.338)	0.584	NA	NA
Maximum tumor diameter (cm)				
≤5	Reference			
>5	1.509 (0.994–2.290)	0.054	1.637(1.075–2.493)	0.022
No. of segments involved				
1	Reference			
≥2	1.508 (0.942–2.413)	0.087	NA	NA
Liver cirrhosis				
No	Reference			
Yes	0.868 (0.561–1.341)	0.523	NA	NA
Poor cell differentiation				
No	Reference			
Yes	1.491 (0.993–2.238)	0.054	1.678(1.113–2.531)	0.014
HBV infection				
HbsAg (−) HbeAg (−)	Reference			
HbsAg (+) HbeAg (−)	0.857 (0.482–1.523)	0.599	NA	NA
HbsAg (+) HbeAg (+)	0.948 (0.438–2.051)	0.891	NA	NA
ALT (U/L)				
≤40	Reference			
>40	1.257 (0.840–1.880)	0.266	NA	NA
AST (U/L)				
≤35	Reference			
>35	1.100 (0.730–1.657)	0.649	NA	NA
Albumin (g/L)				
<40	Reference		Reference	
40–55	0.525 (0.305–0.905)	0.020		
>55	0.719(0.443–1.168)	0.183		
Globulin (g/L)				
≤35	reference			
>35	1.249 (0.815–1.915)	0.307	NA	NA
TBIL (μmol/L)				
≤20.4	Reference			
>20.4	1.056(0.623–1.791)	0.839		
Platelet count (×10^9^/L)				
< 101	Reference			
101–320	1.038 (0.502–2.149)	0.919		
> 320	0.869 (0.315–2.398)	0.787		
PLR				
≤82.4	Reference			
>82.4	1.561 (0.634–3.844)	0.333		
LMR				
≤3.2	Reference			
>3.2	1.605 (0.777–3.318)	0.201		
NLR				
≤6.4	Reference			
>6.4	1.645 (1.094–2.473)	0.017		
APPRI				
≤0.6	Reference		Reference	
>0.6	1.933 (1.261–2.965)	0.003	2.093 (1.359–3.223)	0.001
APRI				
≤0.8	Reference			
>0.8	1.888 (1.207–2.954)	0.005		
PT (s)				
≤13.5	Reference			
>13.5	1.249 (0.648–2.408)	0.506		
AFP (ng/ml)				
<20	Reference			
20–200	1.284 (0.746–2.210)	0.367		
>200	1.354 (0.843–2.175)	0.210		
Child‐Pugh grade				
A	Reference			
B	1.828 (0.944–3.542)	0.074		
Tumor stage				
IA	Reference			
IB	0.922 (0.374–2.274)	0.860		
ALBI				
≤ − 2.6	Reference			
> − 2.6	1.494 (0.954–2.339)	0.080		

Abbreviations: ALT, glutamic‐pyruvic transaminase; AST, glutamic‐oxalacetic transaminase, AFP, alpha fetoprotein; ALBI, albumin‐bilirubin; APPRI, alkaline phosphatase‐to‐platelet count ratio index; APRI, aspartate aminotransferase to platelet ratio index; CI, confidence interval; DFS, disease‐free survival; HCC, hepatocellular carcinoma; HR, hazard ratio; HbsAg/HbeAg, Hepatitis B surface Antigen/Hepatitis be Antigen; HBV, hepatitis B virus; LMR, lymphocytes‐monocytes; MVI, microvascular invasive; NA, not available; NLR = neutrophils‐lymphocytes; PLR, platelet‐lymphocyte; PT, prothrombin time; TBIL, total bilirubin.

**TABLE 2:** Multivariate and univariate analyses of risk factors for overall survival (OS) in patients with hepatocellular carcinoma with microvascular invasion.

	Univariate		Multivariate	
	HR (95% CI)	*p*	HR (95% CI)	*p*
Sex				
Female	Reference		NA	NA
Male	0.748(0.312–1.791)	0.514	NA	NA
Age, years				
≤50	Reference			
>50	1.472 (0.753–2.880)	0.258	NA	NA
Maximum tumor diameter (cm)				
≤5	Reference			
>5	1.213 (0.633–2.327)	0.561	NA	NA
No. of segments involved				
1	Reference			
≥2	1.508 (0.713–3.192)	0.282	NA	NA
Liver cirrhosis				
No	Reference			
Yes	1.162 (0.548–2.466)	0.695	NA	NA
Poor cell differentiation				
No	Reference			
Yes	1.786 (0.943–3.382)	0.075	1.945(1.012–3.738)	0.046
HBV infection				
HbsAg (−) HbeAg (−)	Reference			
HbsAg (+) HbeAg (−)	1.154(0.445–2.991)	0.768	NA	NA
HbsAg (+) HbeAg (+)	1.253 (0.363–4.332)	0.721	NA	NA
ALT (U/L)				
≤40	Reference			
>40	1.585 (0.835–3.006)	0.159	NA	NA
AST (U/L)				
≤35	Reference			
>35	1.468 (0.757–2.845)	0.255	NA	NA
Albumin (g/L)				
<40	reference			
40–55	0.209(0.076–0.574)	0.002		
>55	0.354 (0.165–0.760)	0.008		
Globulin (g/L)				
≤35	Reference			
>35	0968 (0.487–1.924)	0.926	NA	NA
TBIL (μmol/L)				
≤20.4	Reference			
>20.4	1.332 (0.579–3.065)	0.500		
Platelet count (×10^9^/L)				
< 101	Reference			
101–320	0.519 (0.216–1.246)	0.142		
> 320	0.164 (0.024–1.369)	0.095		
PLR				
≤82.4	Reference			
>82.4	1.556 (0.374–6.482)	0.544		
LMR				
≤3.2	Reference			
>3.2	2.497 (0.876–7.111)	0.087		
NLR				
≤6.4	Reference			
>6.4	1.393(0.730–2.655)	0.314		
APPRI				
≤0.6	Reference		Reference	
>0.6	4.523 (2.360–8.669)	<0.001	3.744 (1.669–8.400)	0.001
APRI				
≤0.8	Reference		Reference	
>0.8	4.685 (2.437–9.006)	<0.001	2.824 (1.275–6.255)	0.011
PT (s)				
≤13.5	Reference		Reference	
>13.5	3.602 (1.702–7.627)	0.001	3.858 (1.782–8.354)	0.001
AFP (ng/mL)				
<20	Reference		Reference	
20–200	1.886 (0.680–5.235)	0.223	1.936 (0.689–5.445)	0.210
>200	3.237 (1.385–7.568)	0.007	3.763 (1.565–9.052)	0.003
Child‐Pugh grade				
A	Reference			
B	4.910 (2.078–11.602)	<0.001		
Tumor stage				
IA	Reference			
IB	2.054(0.281–15.022)	0.478		
ALBI				
≤ − 2.6	Reference			
> − 2.6	3.142 (1.560–6.327)	0.001		

Abbreviations: ALT, glutamic‐pyruvic transaminase; AST, glutamic‐oxalacetic transaminase; APPRI, alkaline phosphatase‐to‐platelet count ratio index; APRI, aspartate aminotransferase to platelet ratio index; AFP, alpha fetoprotein; ALBI, Albumin‐Bilirubin; CI, confidence interval; HCC, hepatocellular carcinoma; HR, hazard ratio; HbsAg/HbeAg, hepatitis B surface antigen /hepatitis be antigen; HBV, hepatitis B virus; LMR, lymphocytes‐monocytes; MVI, microvascular invasive; NA, not available; NLR, neutrophils‐lymphocytes; OS, overall survival; PT, prothrombin time; PLR, platelet‐lymphocyte; TBIL, total bilirubin.

### 3.3 Association between APPRI and other clinicopathologic variables

Table 3 shows the comparison of clinicopathologic variables between the APPRI ≤0.74 and APPRI >0.74 groups. The results demonstrated that ALT>40 U/L, AST > 35 U/L, albumin<40 g/L, TBIL >20.4 μmol/L, platelet count<101 × 10^9^/L, NLR >2.52, APRI >0.79, Child‐Pugh grade B, and ALBI >2.6were more common in APPRI >0.74 group (all *p* < 0.05) (Table 3).

**TABLE 3:** Comparison of the clinicopathologic variables between APPRI subgroups.

	All (*n* = 169)	APPRI ≤0.74 (*n* = 122)	APPRI >0.74 (*n* = 47)	*p*
Sex				0.952
Female	22(13.0)	16(13.1)	6(12.8)	
Male	147(87.0)	106(86.9)	41(87.2)	
Age, years				0.585
≤50	74(43.8)	55(45.1)	19(40.4)	
>50	95(56.2)	67(54.9)	28(59.6)	
Maximum tumor diameter (cm)				0.623
≤5	74(43.8)	52(42.6)	22(46.8)	
>5	95(56.2)	70(57.4)	25(53.2)	
No. of segments involved				0.733
1	50(29.6)	37(30.3)	13(27.7)	
≥2	119(70.4)	85(69.7)	34(72.3)	
Liver cirrhosis				0.091
No	52(30.8)	33(27)	19(40.4)	
Yes	117(69.2)	89(73)	28(59.6)	
Poor cell differentiation				0.093
No	109(64.5)	74(60.7)	35(74.5)	
Yes	60(35.5)	48(39.3)	12(25.5)	
HBV infection				0.712
HbsAg (−) HbeAg (−)	24(14.2)	18(14.8)	6(12.8)	
HbsAg (+) HbeAg (−)	126(74.6)	89(73)	37(78.7)	
HbsAg (+) HbeAg (+)	19(11.2)	15(12.3)	4(8.5)	
ALT (U/L)				0.038
≤40	90(53.3)	71(58.2)	19(40.4)	
>40	79(46.7)	51(41.8)	28(59.6)	
AST (U/L)				0.0.003
≤35	70(41.4)	59(48.4)	11(23.4)	
>35	99(58.6)	63(51.6)	36(76.6)	
Albumin (g/L)				<0.001
<40	49(29.0)	23(18.9)	26(55.3)	
40–55	56(33.1)	41(33.6)	15(31.9)	
>55	64(37.9)	58(47.5)	6(12.8)	
Globulin (g/L)				0.093
≤35	69(40.8)	45(36.9)	24(51.1)	
>35	100(59.2)	77(63.1)	23(48.9)	
TBIL (μmol/L)				0.012
≤20.4	136(80.5)	104(85.2)	32(68.1)	
>20.4	33(19.5)	18(14.8)	15(31.9)	
Platelet count (×10^9^/L)				<0.001
< 101	13(7.7)	4(3.3)	9(19.1)	
101–320	143(84.6)	105(86.1)	38(80.9)	
> 320	13(7.7)	13(10.7)	0(0)	
PLR				0.354
≤82.4	13(7.7)	8(6.6)	5(10.6)	
>82.4	156(92.3)	114(93.4)	42(89.4)	
LMR				>0.999
≤3.2	158(93.5)	114(93.4)	44(93.6)	
>3.2	11(6.5)	8(6.6)	3(6.4)	
NLR				0.002
≤6.4	93(55)	76(62.3)	17(36.2)	
>6.4	76(45)	46(37.7)	30(63.8)	
APRI				<0.001
≤0.8	129(76.3)	109(89.3)	20(42.6)	
>0.8	40(23.7)	13(10.7)	27(57.4)	
PT (s)				>0.999
≤13.5	152(89.9)	110(90.2)	42(89.4)	
>13.5	17(10.1)	12(9.8)	5(10.6)	
AFP (ng/mL)				0.994
<20	55(32.5)	40(32.8)	15(31.9)	
20–200	43(25.4)	31(25.4)	12(25.5)	
>200	71(42)	51(41.8)	20(42.6)	
Child‐Pugh grade				0.048
A	156(92.3)	116(95.1)	40(85.1)	
B	13(7.7)	6(4.9)	7(14.9)	
Tumor stage				0.467
IA	10(5.9)	6(4.9)	4(8.5)	
IB	159(94.1)	116(95.1)	43(91.5)	
ALBI				<0.001
≤ − 2.6	122(72.2)	98(80.3)	24(51.1)	
> − 2.6	47(27.8)	24(19.7)	23(48.9)	

Abbreviations: ALT, glutamic‐pyruvic transaminase; AST, glutamic‐oxalacetic transaminase; APPRI, alkaline phosphatase‐to‐platelet count ratio index; APRI, aspartate aminotransferase to platelet ratio index; AFP, alpha fetoprotein; ALBI, Albumin‐Bilirubin; HbsAg/HbeAg, hepatitis b surface antigen/hepatitis be antigen; HBV, hepatitis B virus; LMR, lymphocytes‐monocytes; NLR, neutrophils‐lymphocytes; NA, not available; PT, prothrombin time; PLR, platelet‐lymphocyte; TBIL, total bilirubin.

### 3.4 Kaplan–Meier survival analysis

**FIGURE 2:**
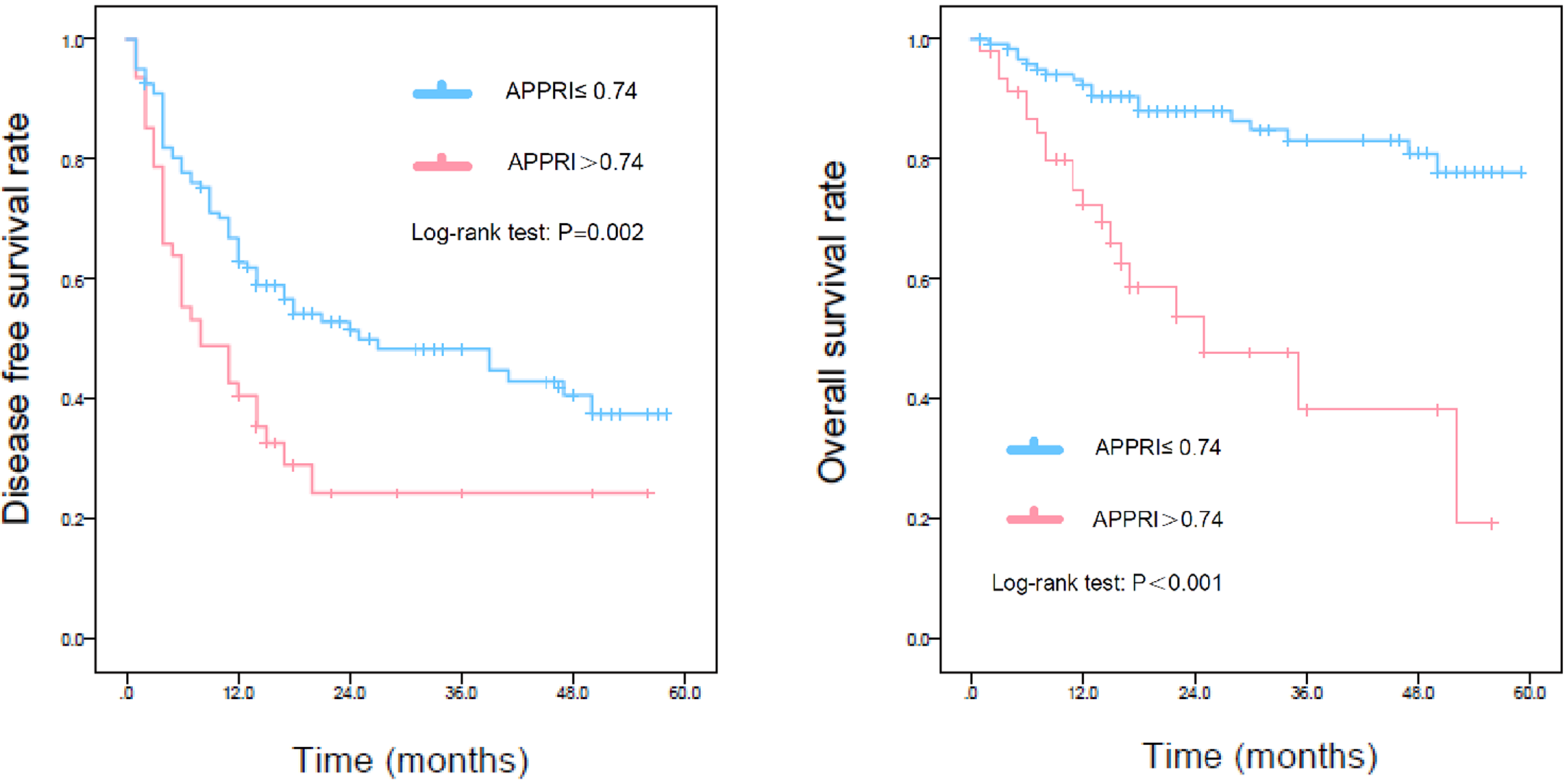
Kaplan–Meier estimates of the prognosis of patients with hepatocellular carcinoma with microvascular invasion. (A) Disease‐free survival curves for patients with APPRI ≤0.74 and APPRI >0.74; (B) Overall survival curves for patients with APPRI ≤0.74 and APPRI >0.74.

To assess the prognosis‐predictive significance of preoperative APPRI in post‐surgical HCC with MVI, a Kaplan–Meier survival analysis was performed. The findings revealed that an APPRI > 0.74 was linked to a significantly shorter OS (Figure 2B) and DFS (Figure 2A). The median DFS time in patients with APPRI >0.74 (8 months, 95% CI: 2.4–13.6) was significantly shorter than that in patients with APPRI ≤0.74 (25 months, 95% CI: 7.1–42.9) (*p* = 0.002) (Figure 2A). DFS rates over 1, 3, and 5 years were 66.90%, 48.40%, and 37.40% for patients with APPRI ≤0.74 and 40.40%, 24.20% and 24.20% for those with APPRI > 0.74, respectively. There was a significant difference in the median OS time of patients with APPRI > 0.74 and APPRI ≤ 0.74 (*p* < 0.001) (Figure 2B) with the former being shorter. Patients diagnosed with APPRI ≤0.74 had 1‐, 3‐, and 5‐year OS rates of 92.40%, 88.10%, and 77.70%, respectively; those diagnosed with APPRI >0.74 had 1‐, 3‐, and 5‐year OS rates of 72.30%, 38.20%, and 19.10% correspondingly. APPRI > 0.74 patients had a remarkably shorter OS and DFS than those with APPRI ≤0.74 (Log‐rank test *p* = 0.002 and *p* < 0.001, respectively).

## 4 DISCUSSION

For patients with HCC and MVI, maximum tumor diameter, poor cell differentiation, and APPRI were independent predictors for DFS and poor cell differentiation, APRI, APPRI, PT, and AFP were independent prognostic factors for OS. However, only platelet‐based APPRI remained an independent predictor of both poor DFS and OS. Patients with APPRI > 0.74 showed considerably shortened DFS and OS duration than those with APPRI ≤ 0.74.

Most of the prognostic factors associated with recurrence after hepatectomy were also associated with recurrence after liver transplantation.^24^, ^25^, ^26^ Adolescent histopathologic factors, such as large tumor volume and poorly differentiated tumors, are predictors of recurrence after hepatectomy and liver transplantation. Poorly differentiated tumors have a poor relapse‐free survival rate and long‐term survival rate, resulting in a worse prognosis for HCC patients following treatment.^27^ HCC is a heterogeneous tumor with different histological types within the same tumor. Studies have shown that the worst histologic grade in the histopathology of advanced HCC may determine the prognosis after radical resection of HCC.^28^ In this research, we discovered that histopathological grade of poorly differentiated tumors was also an independent risk factor for DFS and OS in patients with primary HCC with positive MVI.

Inflammation and angiogenesis are two biomarkers of cancer, which play an important role in all stages of its progression.^29^ The majority of the angiogenic processes are related to inflammation.^30^ Angiogenesis and inflammation are known to interact.^30^ Inflammation promotes tumor genesis and development by changing the tissue microenvironment and further enhancing tumor invasion, migration, and tumor angiogenesis in the microenvironment after inflammatory infiltration.^31^, ^32^ Tumor‐induced systemic inflammatory factors PLR and NLR are prognostic indicators of HCC regardless of the MVI status.^33^, ^34^, ^35^, ^36^ However, the present study demonstrated that PLR and NLR were not significant predictors of OS and DFS in HCC individuals with MVI.

We developed several platelet‐based inflammatory markers by integrating platelet and other hematologic components of the systemic inflammatory factors. Platelets are involved in the growth, invasion, and angiogenesis of tumors.^37^ These are associated with tumor proliferation and metastasis, which help tumor cells survive in metastatic sites.^38^ New blood vessel formation is critical to the biological behavior of solid tumors.^39^ Because they contain over 30 essential proteins and over 80% of the circulating vascular endothelial growth factor, platelets play a pivotal role in the modulation of angiogenesis.^40^ Furthermore, platelets' regulatory roles in inflammation are being widely recognized. Platelets regulate inflammatory responses in cancer by recruiting leukocytes to metastatic and primary tumor sites, as also to the distant organs unaffected by tumor progression, and by altering the activation state of the endothelium.^41^ Several malignancies exhibit an inverse relationship between thrombocytosis and survival.^42^, ^43^, ^44^, ^45^, ^46^, ^47^, ^48^ Nevertheless, some studies have found that low preoperative platelet count indicates poor survival in HCC patients after hepatic resection. In this study, the platelet count was not a significant prognostic factor in patients with HCC and MVI. In light of MVI being a well‐established risk factor of adverse prognosis in HCC, this index alone may be no longer a powerful marker to further stratify HCC patients with MVI. Therefore, our study introduced an index APPRI that was calculated based on the ratio of platelet count and another laboratory parameter, ALP. As a marker of liver injury, ALP is also a tumor‐associated antigen, which reflects the proliferation of cancer cells through nucleoli.^49^, ^50^ The increase in ALP activity in nucleoli is associated with tumor proliferation and progression in the cell cycle. ALP is thought to be an inflammatory marker in cancer. It is an important prognostic predictor for local recurrence and distant metastasis in malignant tumors.^51^, ^52^ Elevated serum ALP predicts worse survival in cancer patients.^32^, ^53^ Interestingly, APPRI was the only independent biomarker for predicting both OS and DFS in HCC patients with MVI, which was in line with a previous study.^20^ Using a cutoff value of 0.74, HCC patients with MVI could be stratified into two subgroups with significantly different survival outcomes.

We also constructed another platelet‐based marker APRI by combing AST and platelets. APRI is an easily measured indirect biomarker used to noninvasively evaluate liver fibrosis and predict the prognosis of patients with chronic hepatitis.^31^ Not only is APRI a robust discriminative index for predicting cancer recurrence and patient survival but it may also indicate the efficacy of a treatment.^19^, ^20^, ^21^ For patients with HBV‐associated small solitary HCC after stereotactic body radiotherapy, APRI is a favorable independent prognostic factor for PFS and OS.^19^ Shi et al. demonstrated that APRI >1.5 is an independent risk factor for liver failure after hepatectomy.^54^ DFS and OS in HCC patients after surgical resection were also observed to be independently linked to APRI >1.68.^21^ Variables including tumor size, population, and sample size all play a role in determining the APRI cutoff value. In this current study, we observed that APRI >0.79 was suggestive of poor OS and DFS in patients with HCC and MVI.

### 4.1 Study limitations

There are certain limitations to this research that need to be mentioned. First, the study was retrospective, which elevated the possibility of selection bias. Second, the sample size was limited, thus, it would be necessary to verify the predictive value of APPRI levels in a larger group of HCC patients. Third, since we only looked at a single HCC, our results may not apply to multiple tumors. Finally, these findings could only be utilized to predict the prognosis for patients with MVI after surgery.

## 5 CONCLUSION

In conclusion, this investigation showed that patients with HCC and MVI who had elevated platelet‐based inflammatory indices, in particular, APPRI, had a worse prognosis. For patients with single HCC and MVI following liver transplantation or resection, preoperative APPRI levels can be used as a noninvasive, simple, and readily measurable prognostic biomarker. Postoperative adjuvant antiplatelet treatment in HCC with increased preoperative platelets needs more clinical studies to determine its effectiveness.

## AUTHOR CONTRIBUTIONS

**Yongxin Zhang:** Conceptualization (lead); data curation (equal); formal analysis (equal); funding acquisition (equal); investigation (equal); methodology (lead); resources (equal); supervision (equal); validation (supporting); writing – original draft (lead); writing – review and editing (equal). **Bin Zhang:** Conceptualization (equal); data curation (lead); formal analysis (equal); funding acquisition (equal); methodology (equal); resources (equal); software (equal); validation (equal); writing – original draft (equal); writing – review and editing (equal). **Lianggeng Gong:** Conceptualization (equal); data curation (equal); formal analysis (lead); funding acquisition (supporting); investigation (supporting); resources (equal); software (supporting); validation (equal); writing – original draft (equal); writing – review and editing (equal). **Liangxia Xiong:** Conceptualization (supporting); data curation (equal); formal analysis (supporting); investigation (equal); project administration (supporting); resources (supporting); software (supporting); validation (equal); writing – original draft (supporting). **Xuehong Xiao:** Investigation (supporting); methodology (equal); project administration (supporting); resources (equal); software (equal); supervision (supporting); validation (equal); visualization (equal). **Chao Bu:** Investigation (supporting); methodology (equal); project administration (supporting); resources (equal); software (equal); supervision (supporting); validation (equal). **Zhiying Liang:** Investigation (supporting); methodology (equal); resources (supporting); software (equal); supervision (supporting); validation (equal); visualization (supporting). **Liangcai Li:** Investigation (supporting); methodology (supporting); resources (supporting); software (equal); supervision (equal); validation (equal); visualization (equal). **Binghang Tang:** Investigation (equal); methodology (supporting); project administration (equal); resources (equal); software (equal); supervision (equal); validation (supporting); visualization (equal). **Yangbai Lu:** Conceptualization (equal); data curation (equal); formal analysis (equal); funding acquisition (lead); investigation (equal); methodology (supporting); project administration (lead); resources (supporting); software (supporting); supervision (supporting); validation (supporting); writing – original draft (equal); writing – review and editing (lead).

## FUNDING INFORMATION

We acknowledge financial support from the National Natural Science Foundation of China (81,871,323 and 81,801,665); the National Natural Science Foundation of Guangdong Province (2018B030311024); the Scientific Research Cultivation and Innovation Foundation of Jinan University (21620447); and Major Project of Scientific Research Foundation, Zhongshan city people's hospital (No.BG20228542).

## CONFLICT OF INTEREST STATEMENT

There authors declare no conflicts of interest.

## Data Availability

The raw data supporting the conclusions of this article will be made available by the authors, without undue reservation.
